# YES‐associated protein‐regulated Smad7 worsen epithelial barrier injury of chronic sinusitis with nasal polyps

**DOI:** 10.1002/iid3.907

**Published:** 2023-06-14

**Authors:** Xiaocong Jiang, Longlan Shu, Yijun Liu, Yang Shen, Xia Ke, Jie Liu, Yucheng Yang

**Affiliations:** ^1^ Department of Otorhinolaryngology The First Affiliated Hospital of Chongqing Medical University Chongqing People's Republic of China

**Keywords:** CRSwNP, nasal epithelial barrier, Smad7, TGF‐β1, YAP

## Abstract

**Background:**

Chronic rhinosinusitis with nasal polyps (CRSwNP) was potentially due to the epithelial barrier injury. YES‐associated protein (YAP) is a multifunctional transcriptional factor and plays versatile roles in the regulation and maintenance of epithelial barrier in different organs and tissues. The purpose of this study is to elucidate possible effect and mechanism of YAP on the epithelial barrier of CRSwNP.

**Methods:**

Patients were divided into CRSwNP group (*n* = 12) and control group (*n* = 9). The location of YAP, PDZ‐binding transcriptional co‐activator (TAZ), and Smad7 were estimated by immunohistochemistry and immunofluorescence. Meanwhile, the expression of YAP, TAZ, Zona occludens‐1 (ZO‐1), E‐cadherin, and transforming growth factor‐beta1 (TGF‐β1) were detected by Western blot. After primary human nasal epithelial cells were treated with YAP inhibitor, the expression level of YAP, TAZ, ZO‐1, E‐cadherin, TGF‐β1, and Smad7 were measured by Western blot.

**Results:**

Compared with the control group, the protein levels of YAP, TAZ, and Smad7 were significantly upregulated, while TGF‐β1, ZO‐1, and E‐cadherin were downregulated in CRSwNP. YAP and Smad7 demonstrated lower levels, while the expression of ZO‐1, E‐cadherin, and TGF‐β1 rose slightly after YAP inhibitor treatment in primary nasal epithelial cells.

**Conclusions:**

Higher level of YAP may lead to CRSwNP epithelial barrier injury via the TGF‐β1 signaling pathway, and the inhibition of YAP can partially reverse epithelial barrier function.

## Graphical abstract

Higher YES‐associated protein (YAP) level may lead to chronic rhinosinusitis with nasal polyps (CRSwNP) epithelial barrier injury via transforming growth factor‐beta1 (TGF‐β1) signaling pathway and the inhibition of YAP can partially reverse epithelial barrier.

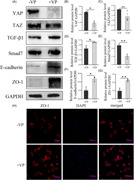

## 1 INTRODUCTION

Chronic rhinosinusitis (CRS) is a condition characterized by ongoing and chronic inflammation of nasal and paranasal sinuses, always lasting over 12 weeks.^1^ It imposes a significant economic burden and negatively impacts the quality of life of patients.^2^, ^3^ For example, CRS has been shown to have a consistent negative impact on cough‐related quality of life and bronchiectasis‐specific quality of life.^4^ CRS can be divided into two phenotypes based on different clinical characteristics: CRS without nasal polyps (CRSsNP) and CRS with nasal polyps (CRSwNP).^5^ Though CRSsNP is more common among patients, standard therapy has greatly improved patient quality of life.^6^ Conversely, CRSwNP patients suffer from much more severe symptoms and complained of a high recurrence rate even after medication therapy and endoscopic sinus surgery (ESS).^1^, ^7^ Consequently, it is important to explore the etiology and pathogenesis of CRSwNP to provide better treatment for the suffering. Recently studies demonstrated that persistent nasal mucosal inflammation stimulation and tissue remodeling could damage the nasal epithelial barrier, which could be essential to the pathogenesis CRSwNP.^8^

The epithelial barrier is the primary fence to protect our body from physical and chemical toxins, allergens, and stimuli in external environment. Many diseases are associated with disruption of the nasal epithelial barrier.^9^, ^10^ The epithelial barrier is mainly composed of epithelial cell junctions, including tight junctions (TJs), adhesive junctions, and desmosomes. Among these, Zona occludens‐1 (ZO‐1) is the most important TJ protein of TJs; and without ZO‐1, the normal function of TJs cannot be formed.^11^, ^12^ Previous studies have shown that the breakdown of nasal epithelial barrier could be potential pathophysiology of CRSwNP.^8^, ^13^, ^14^

Yes‐associated protein (YAP) and PDZ‐binding transcriptional co‐activator (TAZ) are the core effectors of the Hippo pathway. They play a crucial role in organ size regulation and tumorigenesis.^15^, ^16^ They are also associated with various diseases, such as cancer and fibrosis.^17^ Meanwhile, previous studies have evidenced that YAP is involved in maintaining the integrity of the epithelial barrier. For example, in the process of intestinal repair, M2 macrophage‐derived exosomal miR‐590‐3p ameliorated epithelial repair by activating YAP.^18^ In ulcerative colitis, anterior gradient protein 2 activated YAP to suppress TNF‐α‐induced intestinal epithelial barrier injury.^19^ However, the role of YAP and TAZ in epithelial barrier of CRSwNP remains unclear and deserves our attention.

Transforming growth factor‐beta1 (TGF‐β1) is a multifunctional cytokine that typically binds to the TGF‐β1/Smad signaling pathway.^20^ Here, growing evidence implied that TGF‐β1 participated in epithelial barrier function maintenance.^21^, ^22^ For example, in human bronchial epithelial cells, cigarette smoke extract reduced the level of both ZO‐1 and ZO‐2 while higher levels of TGF‐β1 reverse ZO‐1 and ZO‐2 and prevented CSE‐induced TJ destruction and barrier dysfunction. Interestingly, the knockdown of YAP and TAZ reduced TGF‐β1 expression and delayed wound closure in skin wound healing experiments.^23^ Hence, we speculated that there could be an association between the TGF‐β1 signaling pathways and YAP in epithelial barrier.

The purpose of this study was to investigate the role of YAP in the epithelial barrier of nasal polyps, and to preliminarily study the relationship between YAP and the TGF‐β1 signaling pathway in the epithelial barrier of nasal polyps, so as to provide new ideas for the prevention and treatment of nasal polyps.

## 2 METHODS AND MATERIALS

### 2.1 Patients and tissue specimen

Nasal polyps tissue from 12 CRSwNP patients and inferior turbinate (IT) from nine control group patients were harvested during ESS from the Department of Otorhinolaryngology in the First Affiliated Hospital of Chongqing Medical University, China. Control group subjects had no history of CRS who required treatment for nasal septum deviation or repair of cerebrospinal fluid rhinorrhea. Nasal polyp patients were diagnosed with CRSwNP based on the criteria of the European Position Paper on Rhinosinusitis and Nasal Polyps 2020. Accordingly, the exclusion criteria were as follows: immune deficiencies, primary ciliary dyskinesia, fungal sinusitis, cystic fibrosis, antrochoanal polyps, vasomotor rhinitis, and asthma. All enrolled patients did not take any glucocorticosteroids or antibiotics at least 1 month before surgery. Additionally, each individual had no upper or lower respiratory infection and other systemic diseases. Detailed information is shown in Table 1. Informed consent of patients was obtained in this study. This research was approved by the ethics committee of the First Affiliated Hospital of Chongqing Medical University in 2021, and the study approval number is 2021‐107.

**Table 1:** Clinical characteristics of the patients.

	Controls	CRSwNP	P
Sample number	9	12	
Median age (Range)	31 (21–57)	48 (40–56)	NS
Gender (male/female)	5/4	8/4	NS
Smoking history n/N	0/9	0/12	NS
Asthma n/N	0/9	0/12	NS
Allergic rhinitis n/N	0/9	0/12	NS

*Note*: Data presented as medians and interquartile ranges. The level of significance (P) was obtained by Student's *t*‐test. Gender comparison was done by *χ* ^2^ test.

### 2.2 Immunohistochemistry

Tissues were fixed in 4% formaldehyde, embedded in paraffin, and cut into 4 μm per section. These sections were antigen retrieval by buffer solution of citrate, incubated with 3% hydrogen peroxide (Boster Biotechnology) to block endogenous peroxidase activity, and blocked with 10% goat serum to saturate nonspecific staining. Then, sections were incubated with primary antibodies of YAP (1:240, CST, 14074S), TAZ (1:200, abcam, ab224239), and Smad7(1:100, ZENBIO, 860746) at 4°C overnight. The next day, slides were washed with PBS, followed by incubation with secondary antibodies at room temperature for 1 h. After DAB treatment, the sections were observed by inverted microscopy. Analysis of average optical density was performed by using ImageJ software.^24^

### 2.3 Primary human nasal epithelial cells (pHNECs) isolation

Nasal polyps (NPs) tissue and IT mucosa were obtained during general anesthesia surgery and immediately transferred to the lab with liquid nitrogen, washed with cold phosphate‐buffered saline containing penicillin and streptomycin to clear secretion, blood and bone. Here, we isolated pHNECs from tissues by collagenase digestion.^25^ First, tissues were cut into small pellets with scissor, digested with 0.25% pancreatin enzyme for a quarter, and incubated with 0.1% collagenase IV (Sigma, V900893) at 37°C for half an hour. After centrifuged at 4°C for 5 min, liquid supernatant was removed and cell suspension was resuspended with Primary Epithelial Cell Culture System (iCell, PriMed‐iCell‐001). The resuspended cells were cultured in an incubator at 37°C and 5% CO_2_. When pHNECs reached approximately 80% confluence, cells were harvested for further research.

### 2.4 Western blot

Nasal polyps and pHNECs were lysed with RIPA lysate (Beyotime, P0013B) and 1 mM phenylmethylsulfonyl fluoride (Beyotime, ST505) to obtain total protein and protein concentration was measured by BCA protein assay kit (Beyotime, P0012S). After heat denaturation for 15 min at 100°C, 30 μg total protein was electrophoresed on an 8% SDS‐PAGE gel and transferred to a polyvinylidene difluoride membranes (Millipore, IPVH10100). Then, the membranes were blocked with 5% skim milk at room temperature for 1 h. Primary antibodies including TGF‐β1(1:1000, CST, 3711), TGF‐βRII (1:1000, wanleibio, WL02714), Smad2/3(1:1000, CST, 5678S), phospho‐Smad2/3(1:1000, bioss, bs‐8853R), Smad7(1:1000, ZENBIO, 510886/860746), YAP(1:1000, CST, 14074 S), TAZ (1:1000, abcam, ab224239), ZO‐1(1:2000, proteinintech, 21773‐1‐AP), E‐cadherin (1:1000, ZENBIO, 340341), and glyceraldehyde‐3‐phosphate dehydrogenase (GAPDH) (1:3000, ZENBIO, 380626) were diluted with fresh blocking reagent and incubated at 4°C overnight. In the following day, membranes were incubated with secondary antibodies (1:2000) at room temperature for 1 h. Then membranes were washed with PBS for three times, and blots were revealed with enhanced chemiluminescence western‐blotting reagent (ZENBIO, WBULS0500). The photos of the blots were captured and gel documentation of the density of each band was determined using ImageJ software. The ratio of each band/GAPDH was considered as the expression level of the target protein.

### 2.5 Immunofluorescence

The paraffin sections and pHNECs were treated with primary antibodies: YAP (1:300, CST, 14074 S), TAZ (1:100, abcam, ab224239), Smad7 (1:1000, ZENBIO, 510886), and ZO‐1(1:1000, proteintech, 21773‐1‐AP) at 4°C overnight. The next day, slides were incubated with Alexa Fluor 594‐conjugated goat antirabbit secondary antibody for 2 h and nuclei was counterstained with 4,6‐diamidino‐2‐phenylindole. Eventually, images captured by using a confocal laser microscope.

### 2.6 Statiscally analysis

GraphPad Prism 8.0 software and IBM SPSS 22.0 software were used to analyze the data. Two‐tailed Student's *t*‐tests were used to measure *p* value with a *p* < .05 and considered statistically significant. Gender comparison was done by *χ* ^2^ test. All values are presented as the mean ± SD of at least three independent experiments.

## 3 RESULTS

### 3.1 YAP, TAZ, and Smad7 were upregulated in CRSwNP epithelial mucosa

**Figure 1:**
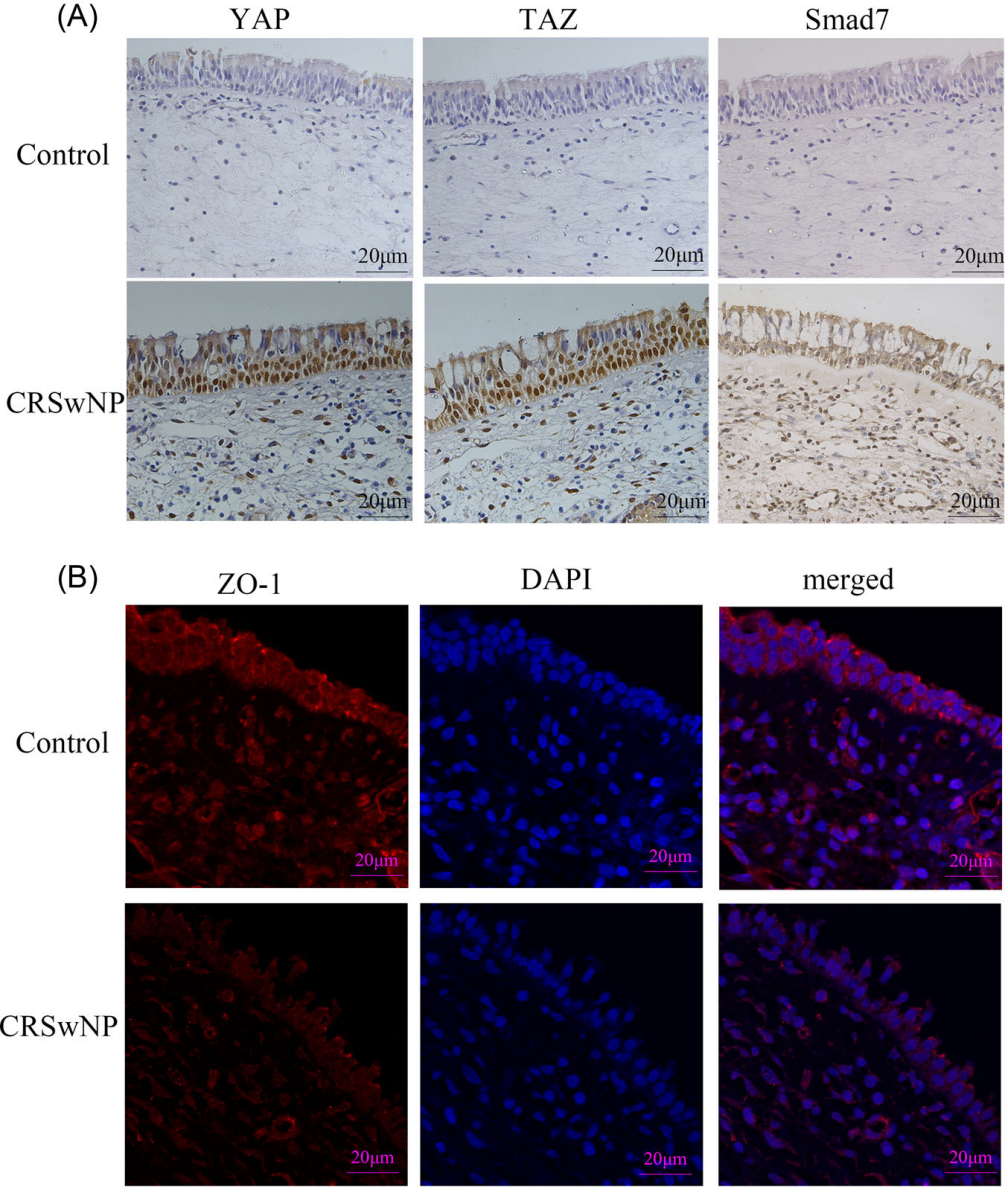
The expression and location of YAP, TAZ, Smad7, and ZO‐1 in CRSwNP. (A) Immunohistochemical staining for YAP, TAZ, and Smad7 in healthy control tissues and CRSwNP tissues. Representative images from each group are shown (*n* = 5, 400× magnification). (B) Immunofluorescence staining for ZO‐1 in healthy control tissues and CRSwNP tissues. Representative images from each group are shown (*n* = 3, 400× magnification). The level of significance (P) was obtained by Student's *t*‐test. CRSwNP, chronic rhinosinusitis with nasal polyps; TAZ, PDZ‐binding transcriptional co‐activator; YAP, YES‐associated protein; ZO‐1, Zona occludens‐1.

To investigate the expression and location of YAP, TAZ, and Smad7 in CRSwNP, we first performed immunohistochemical staining (*n* = 5 for each group). The results (Figure 1A) showed that YAP, TAZ, and Smad7 were higher in CRSwNP group than the control group, and those biomarkers were mainly localized in epithelial mucosa. Moreover, in stark contrast to the control group, the epithelial layer of CRSwNP exhibited an obvious injury. Results (Figure 1B) indicated a lower ZO‐1 expression in the epithelial layers of CRSwNP by immunofluorescence. Overall, the accelerating expression of YAP, TAZ, and Smad7 in epithelial mucosa of CRSwNP was consistent with our speculation about their potential roles in CRSwNP.

### 3.2 YAP/TAZ may damage TJ of epithelium through the TGF‐β1/Smad2/3 signaling pathway

**Figure 2:**
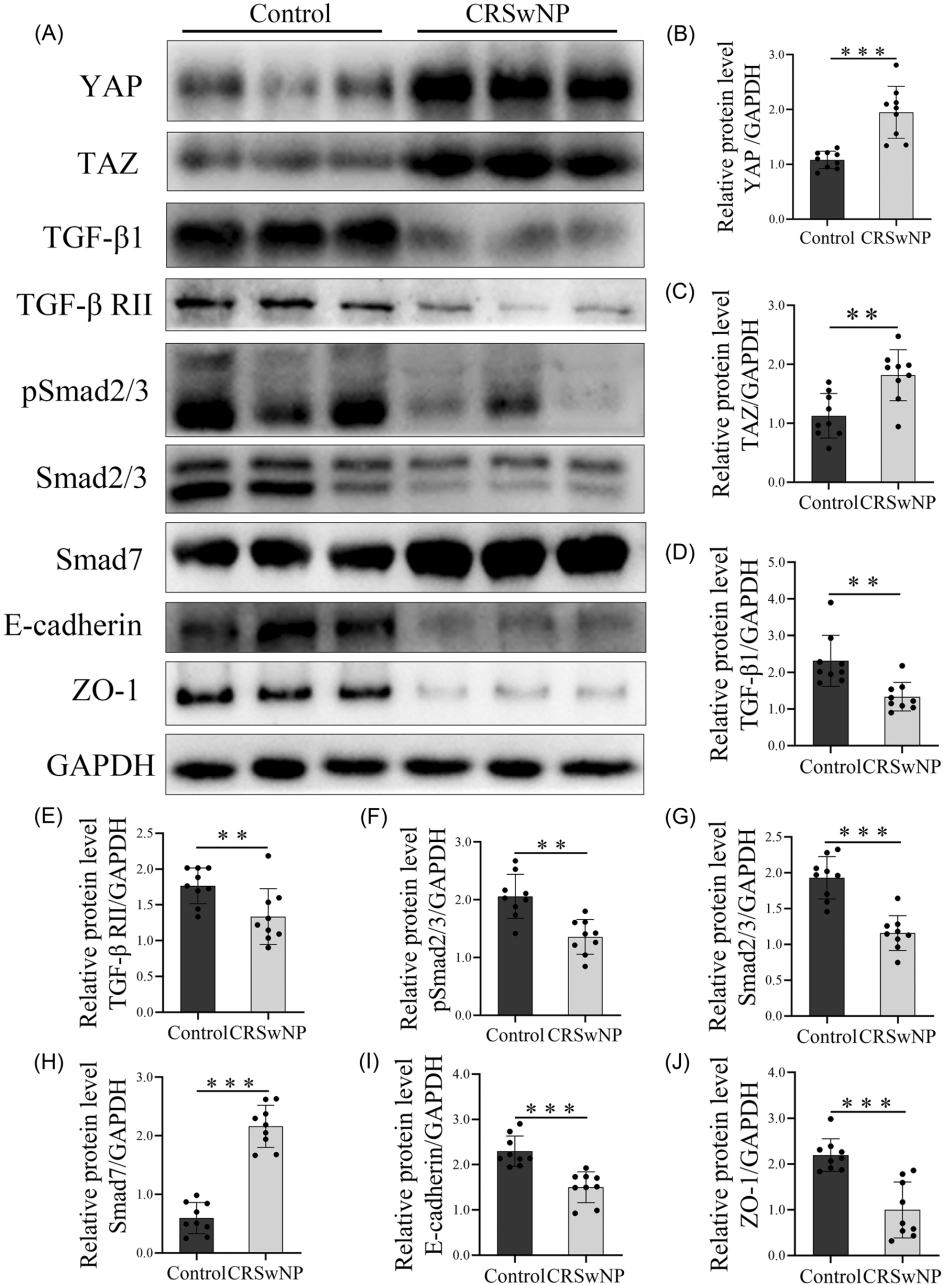
Dysregulated expression of YAP, TAZ, TGF‐β1, Smad7, ZO‐1, and E‐cadherin in CRSwNP tissues. (A) Western blot analysis for YAP, TAZ, TGF‐β1, Smad7, pSmad2/3, Smad2/3, ZO‐1, E‐cadherin, and GAPDH expression in total protein extracted from nasal tissues. Representative images from each group are shown. (B–J) All bands on the panels were quantified by densitometric analysis using ImageJ software, and data were expressed as relative values against GAPDH (*n* = 9). The level of significance (P) was obtained by Student's *t*‐test. *p < .05, **p < .01, ***p < .001. CRSwNP, chronic rhinosinusitis with nasal polyps; GAPDH, glyceraldehyde‐3‐phosphate dehydrogenase; TAZ, PDZ‐binding transcriptional co‐activator; TGF‐β1, transforming growth factor‐β1; YAP, YES‐associated protein; ZO‐1, Zona occludens‐1.

To define the correlation with YAP, TAZ, and TGF‐β1 in TJs, we measured more samples by WB (Figure 2). Compared with normal nasal epithelium, the level of TGF‐β1, TGF‐β RII, and pSmad2/3 were remarkably lower in high YAP/TAZ‐expressed CRSwNP tissues. Consistently, ZO‐1 and E‐cadherin showed a similar trend. Therefore, these implied that YAP/TAZ may damage epithelium TJ through TGF‐β1/Smad2/3 signaling pathway.

### 3.3 YAP and TAZ were mainly expressed in the CRSwNP epithelial cell nucleus

**Figure 3:**
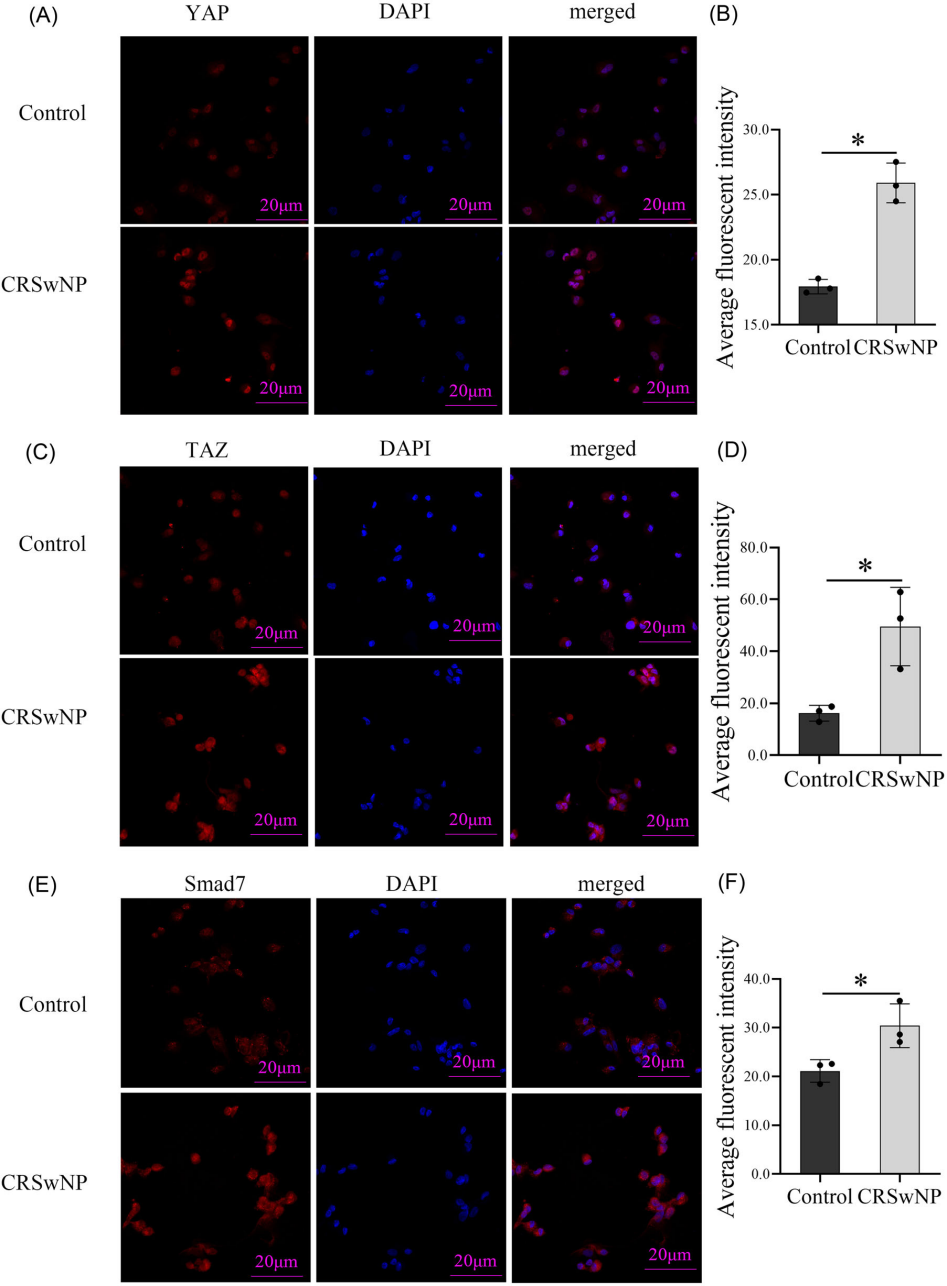
Confocal images of primary epithelial cells derived from CRSwNP tissues and healthy control tissues. Representative confocal images from each group are shown (400× magnification, *n* = 3) The level of significance (P) was obtained by Student's *t*‐test. *p < .05. CRSwNP, chronic rhinosinusitis with nasal polyps; DAPI, 4,6‐diamidino‐2‐phenylindole.

To investigate the expression and localization of YAP, TAZ, and Smad7 in epithelial cells, immunofluorescence staining was applied in next experiment. The results (Figure 3) displayed that YAP and TAZ were expressed in the cytoplasm and nucleus of CRSwNP epithelial cells, but mainly in the nucleus. Additionally, Smad7 was primarily expressed in cytoplasm, but the average fluorescence intensity of CRSwNP group was significantly higher than that of control group.

### 3.4 YAP, TAZ, and Smad7 were upregulated in epithelial cells of CRSwNP

**Figure 4:**
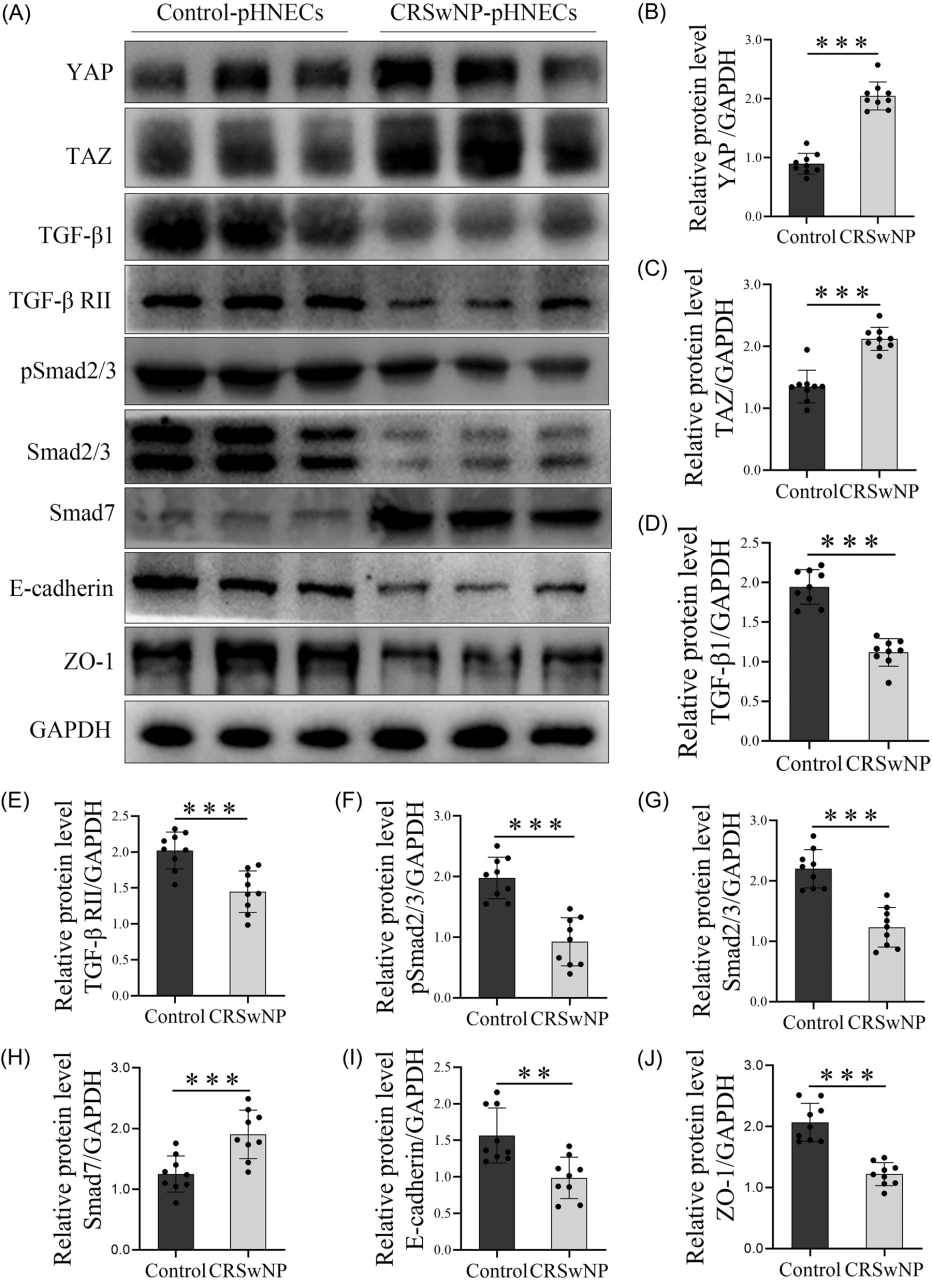
Dysregulated expression of YAP, TAZ, TGF‐β1, Smad7, ZO‐1, and E‐cadherin in CRSwNP epithelial cells. (A) Western blot analysis for YAP, TAZ, TGF‐β1, Smad7, pSmad2/3, Smad2/3, ZO‐1, E‐cadherin, and GAPDH expression in total protein extracted from epithelial cells. Representative images from each group are shown. (B–J) All bands on the panels were quantified by densitometric analysis using ImageJ software, and data were expressed as relative values against GAPDH (*n* = 9). The level of significance (P) was obtained by Student's *t*‐test. *p < .05, **p < .01, ***p < .001. CRSwNP, chronic rhinosinusitis with nasal polyps; GAPDH, glyceraldehyde‐3‐phosphate dehydrogenase; pHNECs, primary human nasal epithelial cells; TAZ, PDZ‐binding transcriptional co‐activator; TGF‐β1, Transforming growth factor‐β1; YAP, YES‐associated protein; ZO‐1, Zona occludens‐1.

We verified the expression of YAP, TAZ, TGF‐β1, and ZO‐1 in nasal polyp‐derived epithelial cells by WB. The results (Figure 4) showed that the expression of YAP, TAZ, and Smad7 in CRSwNP epithelial cells was notably increased. On contrast, the expression of TGF‐β1, TGF‐βRII, pSmad2/3, ZO‐1, and E‐cadherin decreased. This indicated that YAP, TAZ, and TGF‐β1 may participate in epithelial barrier injury by regulating ZO‐1.

### 3.5 Inhibition of YAP can reverse the nasal epithelial barrier

**Figure 5:**
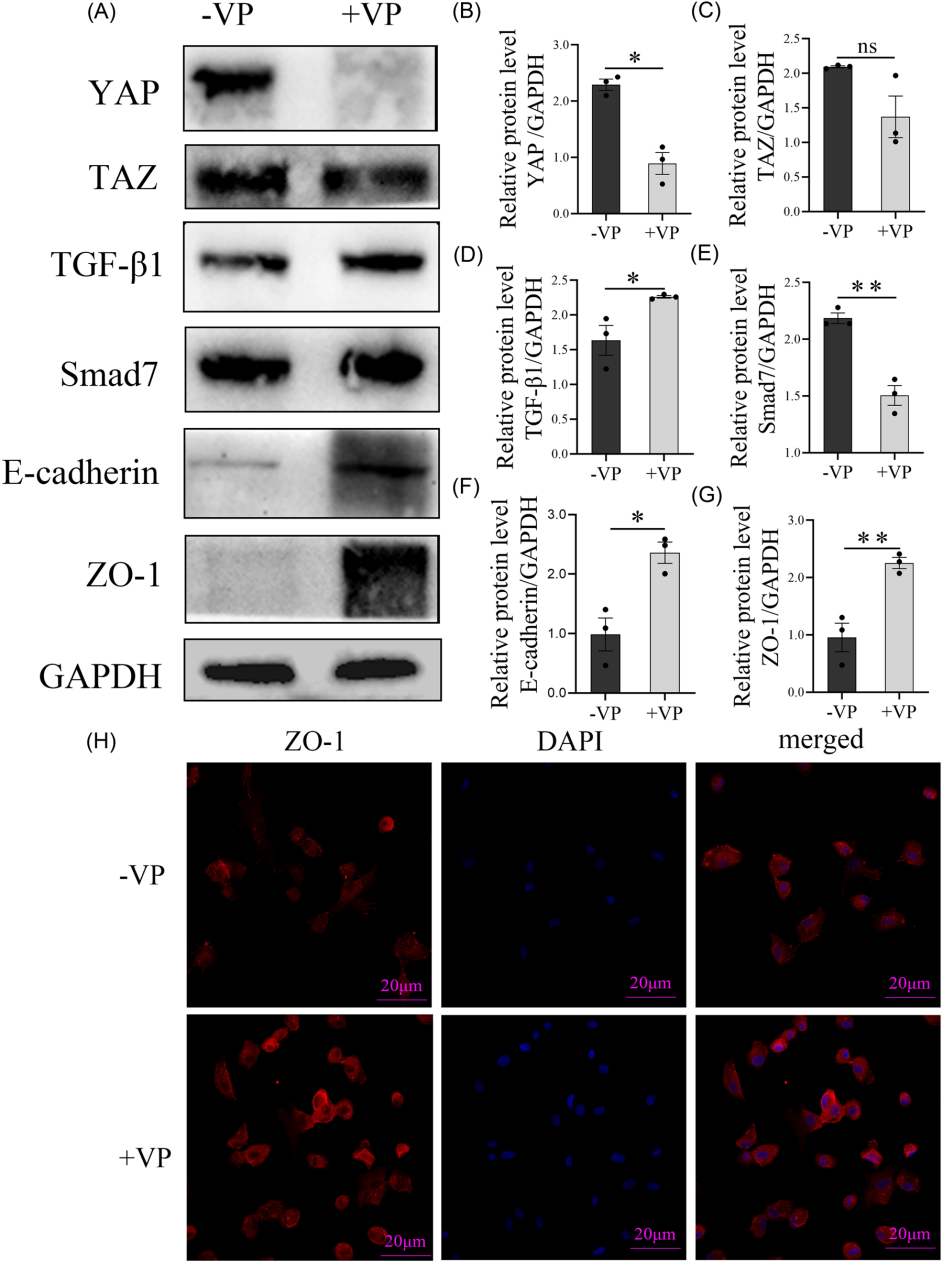
Pharmaceutical inhibition of YAP activity restore CRSwNP epithelial barrier. (A) Protein levels of YAP, TAZ, TGF‐β1, Smad7, E‐cadherin, ZO‐1, and GAPDH from primary nasal polyp epithelial cells stimulated with VP for 12 h. (B–G) All bands on the panels were quantified by densitometric analysis using ImageJ software, and data were expressed as relative values against GAPDH (*n* = 3). (H) Immunofluorescence staining for ZO‐1 in CRSwNP epithelial cells. Representative images from each group are shown (400× magnification, *n* = 3). The level of significance (P) was obtained by Student's *t*‐test. *p < .05, **p < .01, ***p < .001. CRSwNP, chronic rhinosinusitis with nasal polyps; GAPDH, glyceraldehyde‐3‐phosphate dehydrogenase; TAZ, PDZ‐binding transcriptional co‐activator; TGF‐β1, transforming growth factor‐β1; VP, verteporfin; YAP, YES‐associated protein; ZO‐1, Zona occludens‐1.

To further explore whether YAP caused epithelial barrier injury, pHNECs treated with verteporfin (YAP inhibitor) was conducted. Results (Figure 5) indicated that YAP level was significantly reduced and the expression level of ZO‐1, E‐cadherin was notably increased after verteporfin treatment in pHNECs. Meanwhile, the expression of Smad7 showed lower level while the level of TGF‐β1 was higher in CRSwNP epithelial cells respectively, indicating the inhibition of YAP can reverse nasal epithelial barrier.

**Figure 6:**
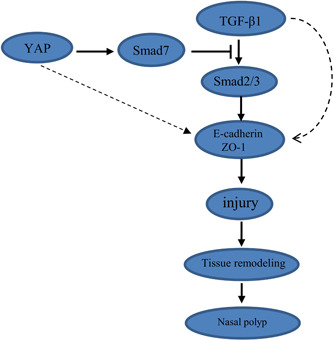
Schematic diagram of the role of YAP in CRSwNP epithelial barrier. CRSwNP, chronic rhinosinusitis with nasal polyps; TGF‐β1, transforming growth factor‐β1; YAP, YES‐associated protein; ZO‐1, Zona occludens‐1.

## 4 DISCUSSION

CRSwNP is a common chronic inflammatory disease in otorhinolaryngology department and patients visit the clinic normally with nasal congestion, decreased smell or anosmia, headache and so on. With increasing levels of pollution, the number of people affected by CRSwNP is on the rise. Therefore, it is urgent to elucidate the mechanism of CRSwNP and provide promising insight into clinical treatment. In this study, we evaluated the different expression of YAP, TAZ, TGF‐β1, TGF‐β RII, pSmad2/3, Smad2/3, Smad7, ZO‐1, and E‐cadherin in CRSwNP and healthy patients. Compared with the control group, the levels of YAP, TAZ, and Smad7 were higher in CRSwNP while TGF‐β1, ZO‐1, and E‐cadherin levels were decreased. After treating pHNECs from NPs with a YAP inhibitor, we observed that the level of Smad7 decreased with increasing levels of ZO‐1, E‐cadherin, and TGF‐β1. These results suggested that YAP may be involved in CRSwNP epithelial barrier injury and the inhibition of YAP could repair nasal epithelial barrier.

Nasal epithelial barrier is the first line to protect our body from being invaded by toxins and stimuli, which is mainly composed of TJs. As one of the most important TJ proteins, the lack of ZO‐1 can lead to abnormal formation of TJ and damage of epithelial barrier. Recent studies revealed that MMP‐10 aggravated podocyte dysfunction and proteinuria by disrupting glomerular filtration integrity via degrading ZO‐1 in chronic kidney disease.^26^ Meanwhile, ZO‐1 was vital in the maintenance of nasal epithelial barrier integrity and its function has been more investigated in allergic rhinitis.^27^ In the current study, the expression of ZO‐1 and E‐cadherin proteins in CRSwNP tissues and epithelial cells was decreased. At the same time, immunofluorescence results showed that the CRSwNP epithelium was significantly damaged compared with the control group, and the expression of ZO‐1 was discontinuous. These results suggested that the epithelial barrier of CRSwNP is impaired, and impaired epithelial barrier may be its potential pathogenesis. Hence, promising direction for the treatment of CRSwNP could be emphasized on the recovery of nasal epithelial barrier in the future.

YAP could take part in the process of epithelial barrier injury and repair. Some research demonstrated that YAP is involved in suppressing TNF‐α‐induced injuries of intestinal epithelial barrier.^19^ Also, some studies found that knockdown of YAP attenuated LPS‐induced airway epithelial barrier dysfunction in 16HBE cells.^28^ However, the role of YAP in CRSwNP epithelial barrier is unclear. This study implied that YAP and TAZ were mainly expressed in the epithelial layer of CRSwNP, and highly expressed in CRSwNP, which was opposite to the expression of epithelial barrier proteins ZO‐1 and E‐cadherin. These results suggested that YAP and TAZ may be related with injuries of CRSwNP epithelial barrier. High expression of YAP may lead to or participate in reducing the expression of TJ protein ZO‐1, eventually leading to epithelial barrier damage. Obviously, further study is needed to address this issue.

Numerous studies examined that TGF‐β1 interacted with YAP and TAZ were involved in many diseases, such as vocal cord fibrosis^29^ and liver fibrosis.^30^ In addition, studies have demonstrated that YAP can bind to Smad7. In HaCaT keratinocytes and COS‐7 cells, YAP binds to Smad7 and enhances the inhibitory activity of Smad7 on TGF‐β signaling pathway.^31^ Tanshinone IIA mediated Smad7 cooperated with YAP to induce apoptosis and inhibit cell and migration by inhibiting TGF‐β signaling pathway in hepatocellular carcinoma cells.^32^ However, the relationship between YAP and TGF‐β1 in the CRSwNP epithelial barrier is unclear. In this study, we indicated that YAP and Smad7 were abnormally highly expressed in CRSwNP while TGF‐β1 and Smad2/3 were lowly expressed. TGF‐β1, pSmad2/3, and Smad2/3 proteins were lowly expressed in CRSwNP tissues and cells, which was consistent with the expression trend of ZO‐1. The endogenous inhibitor Smad7 of TGF‐β signaling pathway was highly expressed in CRSwNP, which was consistent with the expression trend of YAP. Consequently, we speculated that YAP and Smad7 inhibited TGF‐β1 signaling pathway and resulted in epithelial barrier injury. Highly expressed Smad7 may bind to YAP to prevent TGF‐β1 from activating Smad2/3 and inhibiting the expression of TGF‐β1 downstream target genes. Reversing the high expression of YAP and Smad7 may restore the normal expression of ZO‐1 in epithelial cells and repair the epithelial barrier function. These speculations need to be verified by cell experiments.

To further determine the relationship between YAP, Smad7, TGF‐β1, and nasal epithelial barrier injury, we treated CRSwNP epithelial cells with VP which was a potent and selective YAP inhibitor. The results showed that the expression of YAP and Smad7 decreased while TGF‐β1, ZO‐1, and E‐cadherin significantly increased with VP treatment in pHNECs. This indicated that high expression of YAP may contribute to nasal epithelial barrier injury by downregulation of the expression of ZO‐1 and E‐cadherin, while the inhibition of YAP can restore epithelial barrier function by promoting ZO‐1 expression in pHNECs. Interestingly, we also observed a decrease in the level of Smad7 and an increase in TGF‐β1 expression when YAP was inhibited. These results suggest that there may be crosstalk between YAP and TGF‐β1 in CRSwNP, which could be involved in the inhibition of TGF‐β1 signaling pathway by binding Smad7. In brief, our findings indicate that the downregulation of ZO‐1 and E‐cadherin regulated by YAP may contribute to nasal epithelial barrier injury, possibly by inhibiting the TGF‐β1 signaling pathway via Smad7 (Figure 6).

Of course, there are some limitations in this study. First, our investigation was limited to the expression of ZO‐1 without exploring other TJ proteins which could also play a role in epithelial barrier function. Additionally, we did not differentiate between eosinophilic CRSwNP and noneosinophilic CRSwNP, which could have an impact on the results. Further studies should be conducted to address this issue. Last, while our study has provided preliminary evidence for the involvement of YAP, Smad7, and TGF‐β1 in nasal epithelial barrier injury, more detailed investigations are necessary to fully elucidate the underlying mechanisms.

## 5 CONCLUSION

In summary, this experiment verified the expression of YAP in nasal polyps. It was found that the high expression of YAP led to or participated in the decrease of the expression of TJ protein ZO‐1 and participated in the epithelial barrier injury. After inhibiting the expression of YAP, the low expression of ZO‐1 was reversed. At the same time, this study also found that YAP and TGF‐β signaling pathway interact in CRSwNP, but the specific mechanism need to be further explored.

## AUTHOR CONTRIBUTIONS

**Xiaocong Jiang**: Investigation; resources; software; validation; writing—original draft. **Longlan Shu**: Software; supervision. **Yijun Liu**: Software; supervision. **Yang Shen**: Software; supervision. **Xia Ke**: Software; supervision. **Jie Liu**: Software; supervision. **Yucheng Yang**: Funding acquisition; project administration; supervision; writing—review and editing.

## CONFLICT OF INTEREST STATEMENT

The authors declare no conflict of interest.

## Data Availability

The data sets used or analyzed during the current study are available from the corresponding author on reasonable request.
